# Global Spread of the B5 Subgenotype EV-A71 and the Phylogeographical Analysis of Chinese Migration Events

**DOI:** 10.3389/fcimb.2020.00475

**Published:** 2020-09-25

**Authors:** Keqiang Huang, Yong Zhang, Zhenzhi Han, Xiaofang Zhou, Yang Song, Dongyan Wang, Shuangli Zhu, Dongmei Yan, Wen Xu, Wenbo Xu

**Affiliations:** ^1^WHO WPRO Regional Polio Reference Laboratory and National Laboratory for Poliomyelitis, National Health Commission Key Laboratory for Biosafety, National Health Commission Key Laboratory for Medical Virology, National Institute for Viral Disease Control and Prevention, Chinese Center for Disease Control and Prevention, Beijing, China; ^2^Center for Biosafety Mega-Science, Chinese Academy of Sciences, Wuhan, China; ^3^Yunnan Center for Disease Control and Prevention, Kunming, China

**Keywords:** B5 subgenotype, enterovirus A71, molecular evolution, migration, phylogeographical analysis

## Abstract

The subgenotype B5 of EV-A71 is a widely circulating subgenotype that frequently spreads across the globe. Several outbreaks have occurred in nations, such as Malaysia, Thailand, Vietnam, and Japan. Appearing first in Taiwan, China, the subgenotype has been frequently reported in mainland of China even though no outbreaks have been reported so far. The current study reconstructed the migration of the B5 subgenotype of EV-A71 in China via phylogeographical analysis. Furthermore, we investigated its population dynamics in order to draw more credible inferences. Following a dataset cleanup of B5 subgenotype of EV-A71, we detected earlier B5 subgenotypes of EV-A71 sequences that had been circulating in Malaysia and Singapore since the year 2000, which was before the 2003 outbreak that occurred in Sarawak. The Bayesian inference indicated that the most recent common ancestor of B5 subgenotype EV-A71 appeared in September, 1994 (1994.75). With respect to the overall prevalence, geographical reconstruction revealed that the B5 subgenotype EV-A71 originated singly from single-source cluster and subsequently developed several active lineages. Based on a large amount of data that was accumulated, we conclude that the appearance of the B5 subgenotype of EV-A71 in mainland of China was mainly due to multiple migrations from different origins.

## Introduction

The Enterovirus A71 (EV-A71), is a fast-spreading pathogen, and causes hand-foot-and-mouth disease (HFMD), which imposes a serious burden on children and infants, worldwide. Reportedly, it may also cause aseptic meningitis, encephalitis, acute flaccid paralysis, and severe respiratory diseases, among others (Griffiths et al., 2012; Apostol et al., 2019). Although, it is considered that it was isolated first in California in 1967, some evidence presented in 2009 suggest that it may have appeared somewhat earlier in the Netherlands during the 1963–1966 period (Schmidt et al., 1974; Van Der Sanden et al., 2009). Seven genotypes (A–G) and 14 subgenotypes have been detected since the establishment of the molecular typing method of EV-A71 genotype and subgenotype based on entire *VP1* sequences (Laxmivandana et al., 2013; Bessaud et al., 2014; Saxena et al., 2015). Some subgenotypes have been shown to be strongly associated with outbreaks or severe cases (Wang et al., 2002; Ji et al., 2019). In addition to intergroup genetic differences, which have been used as a standard method for genotyping, there appears to be a strong relationship between geographic origins. Therefore, it is possible to identify geographical subgroups even under conditions of ongoing migration.

The subgenotype C4 serves as a good example of localization as well as migration. Moreover, the subgenotype C4, which was first classified in China in 2004, is also an important cluster consisting of re-emerging pathogens (Nagata et al., 2004), and the large scale outbreak in China was found to be associated with “C4a evolutionary branch,” a selective clade of the subgenotype C4 (Zhang et al., 2009; Zhang and Xu, 2013). In fact, this branch has possibly been in existence for 10 years and the C4 subgenotype for 14 years [the time of most recent common ancestor (T_MRCA_) of C4a evolutionary branch: 1998.4; the T_MRCA_ of C4b subgenotype: 1994.1] before the outbreak that resulted in 353 severe cases and 22 fatalities in the Anhui province of China in 2008 (Zhang et al., 2010, 2011). Subsequently, the C4a evolutionary branch has been considered as the predominant type causing HFMD in China, especially leading to severe and fatal cases (Ji et al., 2019). To date, it has spread around the world, and previous studies have held it responsible for outbreaks in Cambodia, Vietnam, and Denmark (Fischer et al., 2014; Duong et al., 2016; Duy et al., 2017).

The B5 subgenotype, which was detected several years after the discovery of C4, is also considered to be a predominant genotype circulating in a manner similar to that of the C4 subgenotype (Huang et al., 2008; Mirand et al., 2015). It was first described in an outbreak that occurred in Sarawak, Malaysia (Podin et al., 2006). Since then, it has caused several outbreaks and persistent epidemics in Singapore, Japan, and Thailand (Wu et al., 2010; Puenpa et al., 2011; Mizuta et al., 2014). Currently, the B5 subgenotype exhibits a stable circulation in south-east Asia and frequently spreads to the neighboring countries.

Most field EV-A71 sequences, as well as many sequences submitted to Genbank, are based on the HFMD case surveillance system data of certain countries from which it is possible to obtain more information on the B5 subgenotype of EV-A71. However, traditional studies associated with the molecular epidemiology of EV-A71 have focused on the evolution and dynamic composition of the subgenotypes, and rarely described systematic spread or changes in population structure. It may be useful to determine whether an imported population played an important role in the dynamic composition of local clusters. One may also perform a risk assessment of migrating strains. Especially in China, it is well-known that the C4a subgenotypes are the localized persistently circulating clades (Zhang et al., 2011). For the other subgenotypes, more evidence needs to be analyzed.

Phylogeographic analysis is used to track migration and make predictions. Previously, it has been widely used for diseases like rabies, influenza, and yellow fever (Beck et al., 2013; Dellicour et al., 2019; Mino et al., 2019). The current study attempted to illustrate the state of spread of the B5 subgenotype of EV-A71. Furthermore, we attempted to understand migratory events in the Yunnan province of China, and explored the relationship between recent migratory events.

## Materials and Methods

### Sample Collection

We collected stool samples from throat swabs and stools of 5-year-old children with diagnosed HFMD at the sentinel hospital in Yunnan Province of China. This study was carried out in accordance with the recommendations of “China national HFMD case surveillance program−2009 edition,” and the protocol used in this study was approved by the Second Ethics Review Committee of the National Institute for Viral Disease Control and Prevention, Chinese Center for Disease Control and Prevention. All subjects gave written informed consent in accordance with the *Declaration of Helsinki*.

### Virus Isolation and *VP1* Sequencing

We processed stool samples according to the standard protocol, and inoculated them on rhabdomyosarcoma cell lines (RD) (Xu and Zhang, 2016). We harvested viruses from RD cell line according to the technical protocol stipulated for China National HFMD case surveillance program (2009 edition). We extracted viral RNA using a QIAmp RNA Mini Kit (Qiagen, USA) and stored them below −20°C. We used specific primers (Zhang et al., 2009) to amplify the entire *VP1* region via a PrimeScript One Step RT-PCR Kit (Takara, Dalian, China). We purified the PCR products using a QIAquick PCR purification kit (QIAgen, Germany) and sequenced them using an ABI 3130 Genetic Analyzer (Applied Biosystems, Foster City, CA, USA) via the dideoxy chain termination method. We assembled both results from each strand using the Sequencher v5.0.

### Dataset and Data Cleaning

We downloaded and integrated all EV-A71 sequences from Genbank and our local database. We aligned each sequence with the entire *VP1* sequence (891 nt) of the prototype strain BrCr (accession number AB204852) and trimmed to the same length of 891 nt. We made a phylogenetic tree with substitution model of Gama-distributed maximum likelihood composite (ML+Γ4) to clearly classify all sequences into different serotypes, and we save all sequences belonging to the B5 subgenotype as a raw dataset using MEGA 6.06 with bootstrap replication set at 1000 (Tamura et al., 2013). We classified the subgenotype sequences was also and verified them via the Enterovirus genotyping pool website (Kroneman et al., 2011).

For the raw dataset, we utilized a non-clock phylogenetic tree and analyzed the regression of root-to-tip distance on sampling time using TempEst v1.5.1 (Rambaut et al., 2016). Concurrently, we prepared a new dataset where the streamlined data was saved in order to reduce the invalid sample size. We focus on sequences with strong epidemic evidence. Scattered data often represents sequences with annotation errors or biases, even if the reduced data may help to find more relationships, including those with low trust. We use both datasets for further analysis.

### Population Status and Dynamic Distribution Inference

We inferred population dynamics via two steps. First, we assessed the dynamics of the B5 subgenotype populations using a Bayesian Skyride method (Drummond et al., 2002, 2005). Simultaneously, we calculated the Tajima's *D*-test and the Fu's test using DNASP version 6 in order to determine the status of the populations (Rozas et al., 2017). Additionally, we used a mismatch distribution method was used to evaluate the population, this involved the use of a curve fitting algorithm via Arlequin v3.5.2 (Excoffier and Lischer, 2010). Second, T_MRCA_ for the whole dataset was reconstructed. We performed a Bayesian associated analysis was performed using the Beast v1.10 packages (Suchard et al., 2018). A constant size model and an uncorrelated lognormal molecular clock were selected. A Jmodel test 2.0 package (Darriba et al., 2012) was used to select the substitution model. The general time reversible model with the Gama-distributed across-site variation (GTR+Γ4) was considered as the best model available in the Beast v1.10 packages, due to the smaller values of the Bayesian information criterion (BIC) and the Akaika information criterion (AIC). We subjected the raw dataset to the same procedures for purposes of comparison and to avoid bias in data selection.

### Recombination Analyses

The *P2* and *P3* coding region sequences of the B5 sub-genotype of EV-A71 genomes were analyzed using the BLAST server to compare their identity with sequences from GenBank. With the similarity of sequences higher than 90%, these sequences were considered potential parents and were downloaded from the GenBank. After screening all potential donors, we used a small dataset of open reading fragment (ORF), including seven genomes identified in this study. The SimPlot program (version 3.5.1) was used to carry out similarity plots and bootscanning analyses, with a 200-nucleotide window moving in 20-nucleotide steps. Bootscanning analyses were run using the neighbor-joining method (Salminen et al., 1995).

### Phylogeographic Reconstruction of B5 Subgenotype

The Monte-Carlo algorithm and the Bayesian statistics provided the framework for investigating migratory events in time and space. We utilized a discrete spatial diffusion model and an uncorrelated lognormal molecular clock model. We implemented the Bayesian stochastic search variable selection (BSSVS) procedure to construct a Bayes factor (BF) test that identifies the most parsimonious description of the phylogeographic diffusion process (Lemey et al., 2009). Briefly, the BSSVS enables the simultaneous determination of the non-zero movement rates among the pairs of locations and efficiently infer the geographical ancestor. In addition, we used the method of calculating the BF to draw migratory inferences. Through BF method, BF value and average posterior value were calculated to infer the possible migration path between the two regions (At least BF >3 and average posterior value >0.5, the migration path is considered to be valid). The migration between regions is measured by the parameter “mean rate” in BSSVS analysis.

We implemented this process, via a well-established temporal-spatial model, via the Beast v1.10 packages together with Beagle (Bielejec et al., 2014). We performed a Markov chain Monte Carlo (MCMC) algorithm for two independent processes of 120 million generations, with sampling occurring every 8,000th generation. We used Tracer v1.5 to diagnose the processes of mixing as also to assess effective sample size (ESS). To be precise, a parameter with an ESS of more than 200 must be considered for the result to be credible.

We annotated a maximum clade credibility (MCC) tree in the Tree Annotator by ignoring the first 1,500 trees, in which nodes' posterior were summarized, and visualization was performed by FigTree. In order to simulate a dynamic process of migration, we converted the MCC tree into a keyhole markup language (KML) file suitable for viewing on Google Earth.

## Results

### Prevalence of the B5 Subgenotype of EV-A71 Is Divided Into Two Stages

**Figure 1:**
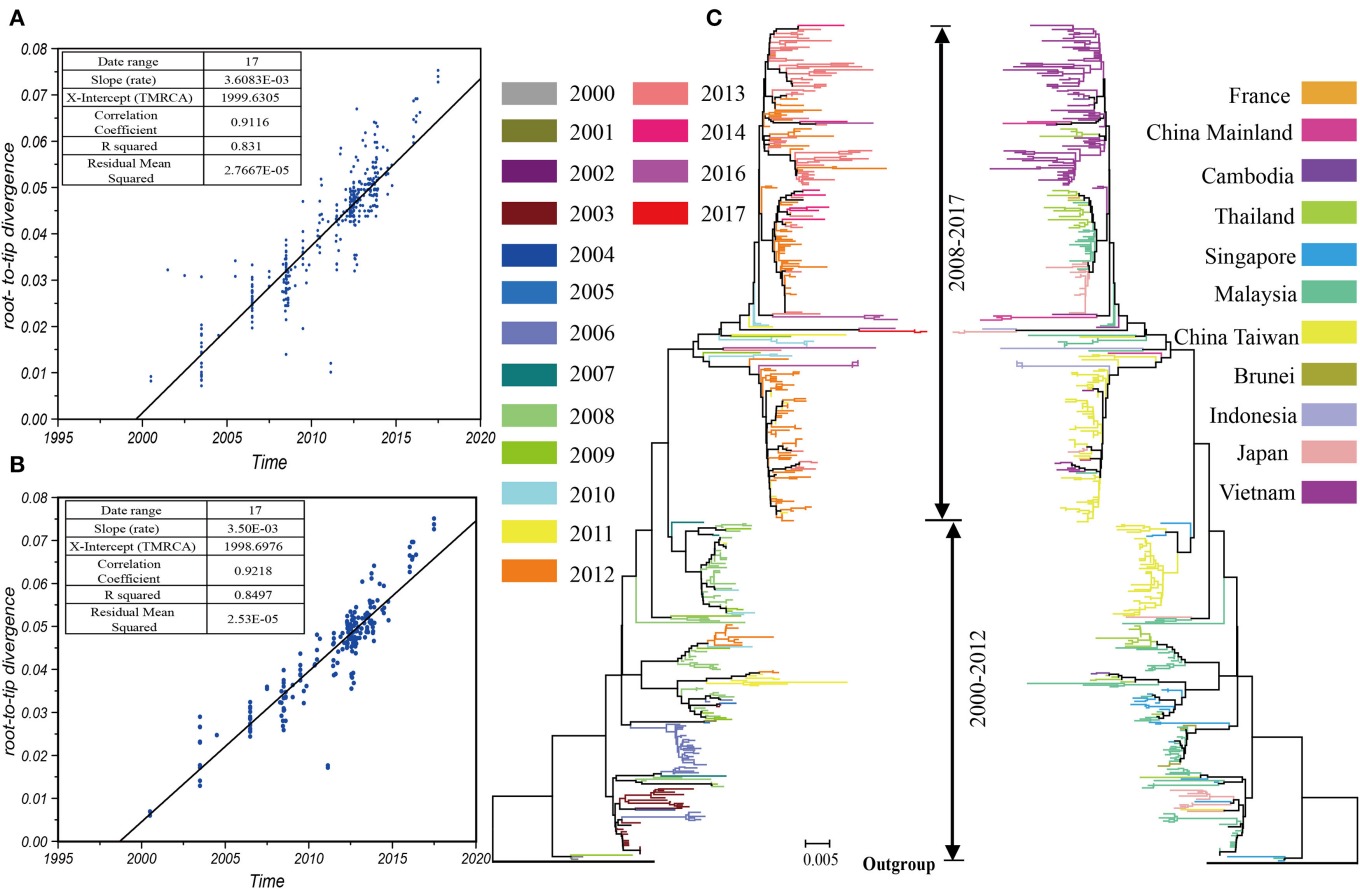
The prevalence of the B5 subgenotype of EV-A71 is divided into two stages. The root-to-tip diagnostic tool using heuristic residual mean square methods suggested that data redundancy and discrete nodes existed in the total dataset **(A)** and the compact dataset **(B)**; the phylogenetic tree indicated the recent epidemics (after 2012) were mostly found in the end of a branch of the evolutionary tree **(C)**.

We collected 519 sequences belonging to the B5 subgenotype from 7,012 sequences containing the entire *VP1* region of EV-A71, of which 5 sequences were identified in the HFMD cases in China. The phylogenetic tree (Figure 1C) of the whole dataset illustrated a large number of sequences identified from past outbreaks. The pairwise distances between these sequences, computed using P-distance models, were commonly <0.005. Epidemics of the B5 subgenotype of EV-A71 were divided into two stages; 2000–2012 and 2008–2017, with an intermediate interchange from 2008–2012. The root-to-tip divergence, which implemented the heuristic residual mean square models, revealed that the datasets were redundant and existed the discrete nodes (Figure 1A). These discrete nodes generally indicate sequence associated issues, such as those related to sample collection dates or sequencing errors. Too many discrete nodes may pose a challenge to the processes of sample mixing as well as the computing of resources. Therefore, we decreased discrete nodes. In total, we analyzed 195 sequences. The status of the submission sequences from 10 countries and regions, through 17 years since 2000, involved in the parsimony dataset has been shown (Figure 1B). The phylogenetic tree (Figure 1C) indicated many clusters here depending on the threshold that was used, however the strains that caused recent epidemics (after 2012) were mostly found in the end of the evolutionary tree. Compared with the first cluster that was simply spread around south-east Asia, the latest cluster was spread more widely and displayed more complex relationships.

### Estimation of T_MRCA_ and Population Dynamics for the B5 Subgenotype of EV-A71

**Figure 2:**
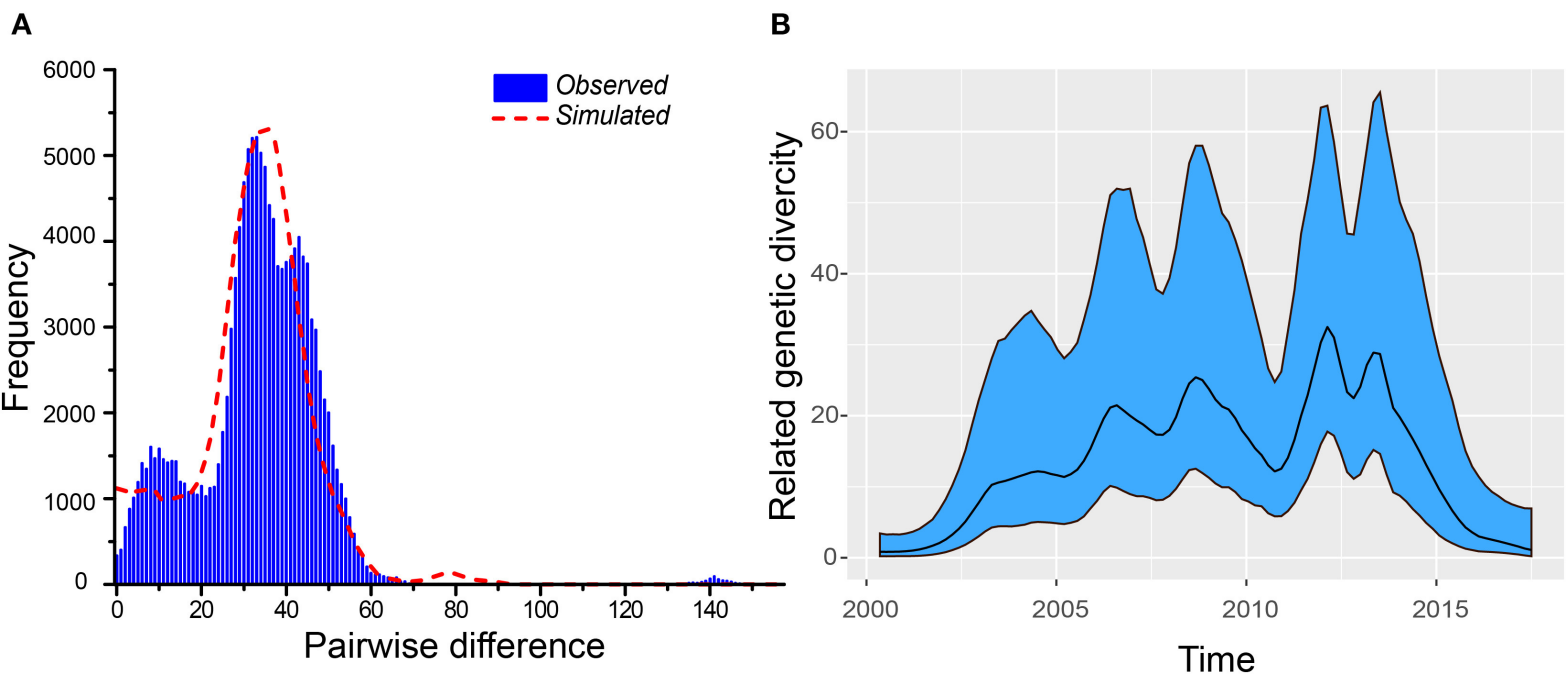
Estimation of T_MRCA_ and population dynamics for the B5 subgenotype of EV-A71. **(A)** The results of the mismatching distribution showing that the simulated curves were in good agreement with the measured ones. **(B)** The Bayesian skyride plot indicates that more expansions occurred.

We analyzed 519 *VP1* sequences belonging to the B5 subgenotype. We used different methods to evaluate the B5 subgenotype population, and most of the methods showed that an expansion event had occurred. Interestingly, although the values of both the Tajiama's test and the Fu's test were negative, no statistical difference was indicated by the Tajiama's test (*P* > 0.05). By contrast, the results of mismatching distribution showed that the simulated curves were in good agreement with measured ones (SSD < 0.01, *R* < 0.01, *P* > 0.05) (Figure 2A). Although this indicates that the population represented by the given dataset at least had two expansions, the actual dynamics may be more complex. The Bayesian skyride plot offers further explanations (Figure 2B). The first expansion appears to have occurred before 2012, even though multiple fluctuations were observed during this period. During 2010–2013, we observed two fluctuations of genetic diversity, followed by the rapidly decreasing till 2017. In conclusion, our study suggests that the population dynamics and their structure may require further phylogeographical analysis.

The T_MRCA_ of the *VP1* sequences illustrated that the B5 subgenotype of EV-A71 may have differentiated in 1994.75 [95% Highest Probability Density (HPD): 1988.66–1998.97] and that it has been in circulation for at least 20 years. The main rate of nucleotide substitution is 5.6 × 10^−3^ [95% HPD: 4.83 × 10^−3^-6.44 × 10^−3^]. Except for a few external nodes, the evolution rate has slightly changed during the epidemic period (Table 1). This may enhance the evaluation of sample quality for further phylogeographical analysis. Although it may not represent the real ancestor of this complex migration process, it may at least identify the earliest ancestor and the first time it appeared.

**Table 1:** The T_MRCA_ of the B5 subgenotypes of EV-A71 in different countries and regions.

**T_**MRCA**_**	**1994.75**	**95%HPD**	**(1988.66, 1998.97)**
Mean rate	5.65 × 10^−3^	95%HPD	(4.83 × 10^−3^, 6.44 × 10^−3^)
Location		T_MRCA_	95%HPD
France		2013.24	(2012.90, 2013.49)
Malaysia		1994.79	(1988.66, 1998.98)
Taiwan, China		1996.27	(1991.19, 2000.39)
Vietnam		1999.1	(1994.61, 2004.52)
Brunei		2003.58	(2000.15, 2005.74)
Cambodia		1998.33	(1993.44, 2001.11)
Mainland of China		1997.49	(1992.87, 2000.53)
Indonesia		2006.47	(2005.49, 2007.36)
Japan		1995.62	(1990.16, 1998.72)
Singapore		1994.77	(1988.66, 1998.96)
Thailand		1997.39	(1992.4, 2000.63)

### Estimation of Geographical Origin and Spread of B5 Subgenotype of EV-A71

**Figure 3:**
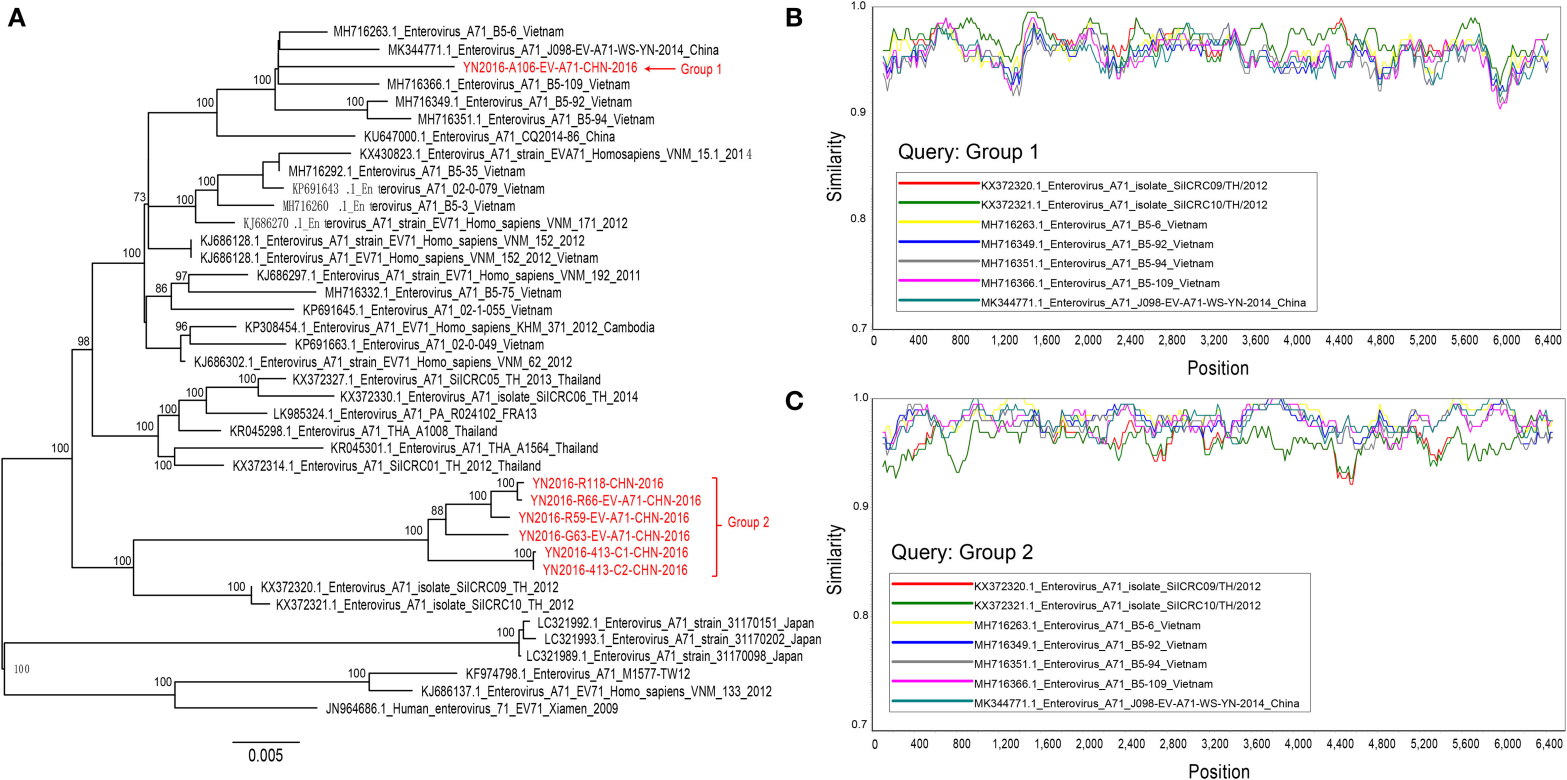
Analyis of recombination events of B5 sub-genotype of EV-A71. **(A)** A maximum likelihood tree (1,000 bootstrap replicates) was constructed, and seven Yunnan B5 sub-genotype of EV-A71 genomes were separated into two groups; **(B,C)** no significant inter-serotypes or inter-species recombination events were found.

A maximum likelihood tree (1000 bootstrap replicates) was constructed, and we found that seven Yunnan B5 sub-genotype of EV-A71 genomes were separated into two groups, and these two groups of viruses are most similar to B5 sub-genotype of EV-A71 from Thailand and Vietnam, respectively (Figure 3A). When we analyze the recombination events of Yunnan B5 sub-genotype of EV-A71 with other B5 sub-genotype of EV-A71 sequences by using seven statistics methods of RDP4.0 and Similarity plot, no significant inter-serotypes or inter-species recombination events were found (Figures 3B,C). The possible reason is that most of the genomic sequences of B5 sub-genotype of EV-A71 were identified in south-east Asia countries, and that the ancestor donors were rarely investigated.

In geographical reconstruction, the BSSVS has a strong impact on the location root. However, many localities that were weakly supported as root locations showed negligible posterior probability under the BSSVS. Interestingly, when Google Earth was used to reveal the dynamic process, and priors of the minimal rate configuration of epidemiological links were considered, 50 branches connecting 16 locations were identified. Migratory processes have been shown (Supplementary Video in Supplemental Material 1). These processes suggest that diffused multiplied migrations may have occurred in mainland of China during the past years. Moreover, so far, no inter-province linkages have been observed except in Taiwan, China. Because of the complexity of its geography and demographics, the B5 subgenotype epidemics in Taiwan are different from those in mainland of China. Many linkages have been shown between Malaysia, Japan, Singapore, and Indonesia. Two migrations have been indicated, one to Xiamen, China in 2009 and the other to Jiangsu, China in 2011.

**Figure 4:**
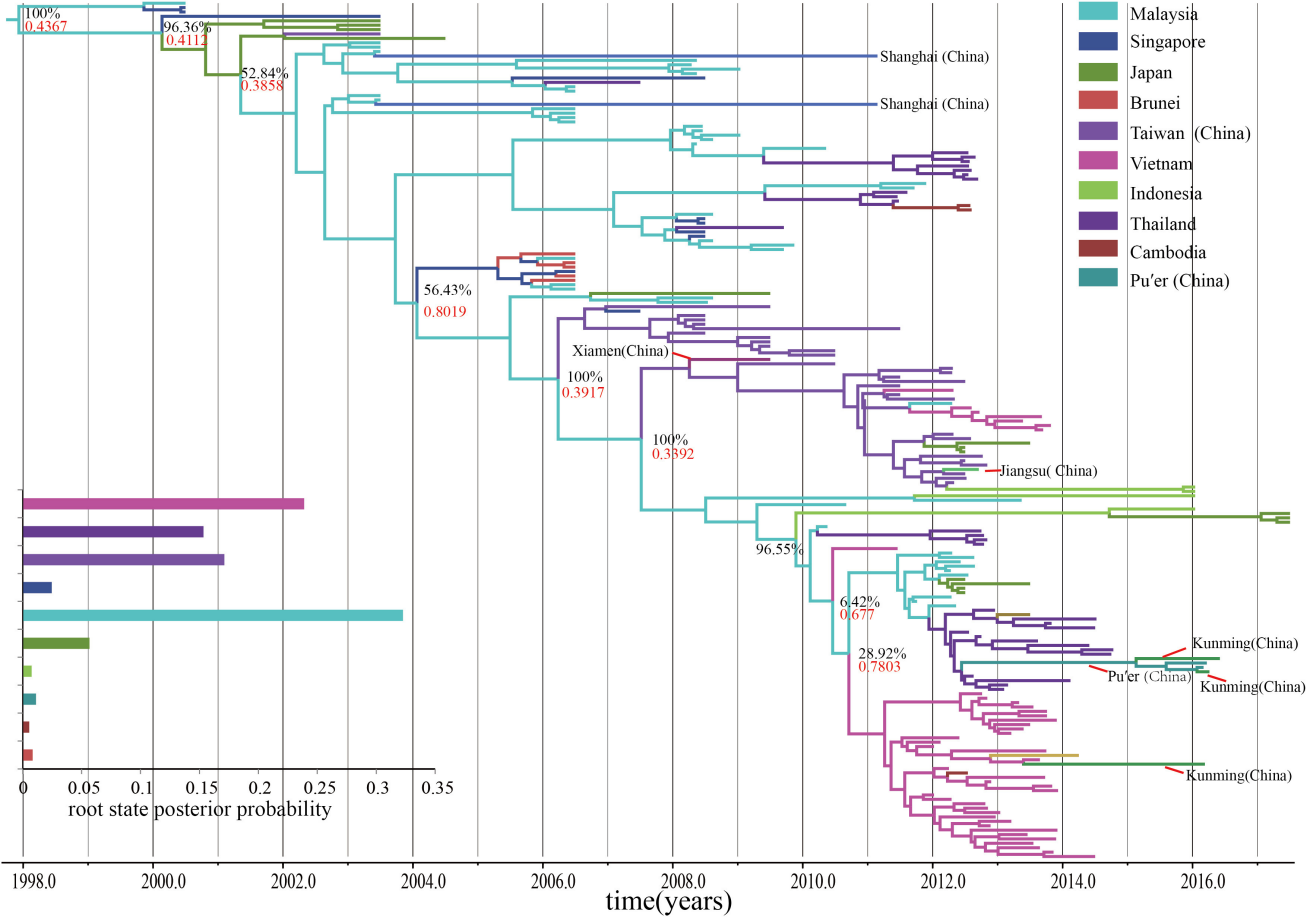
The time-scaled MCC tree constructed based on the entire *VP1* region. The time-scaled MCC tree reveals that all the B5 subgenotypes of EV-A71 were from a geographical mono-originated cluster. Posterior probability, an indicator of phylogeography, shows that continuous processes with particular reference to the connections between Malaysia and other countries, such as Japan, Thailand, and Vietnam.

The time-scaled MCC tree based on the entire *VP1* region showed that all the B5 subgenotypes belonged to a geographical mono-originated cluster (Figure 4). The geographical sub-group of Malaysia played an important role in the subsequent spread of the B5 subgenotypes during the past 20 years. However, the root state posterior probability is not very high (0.4367), and this single-source spread changed after 2007, with multiple origination overserved. Most B5 subgenotypes spreads from other geographical regions, such as Vietnam and Thailand, become popular, even though the Malaysian subgroup continuously play significant role for B5 subgenotypes diffusion. Similar to the statistics of the node status, it indicates that the nodes representing Malaysia, Vietnam, and Thailand shared a vast majority of the posterior mass. In regard to the continuous spread, the process of spread of the subgenotypes among the countries in Southeast Asia as well as in the west pacific region appears to be quite complex.

**Figure 5:**
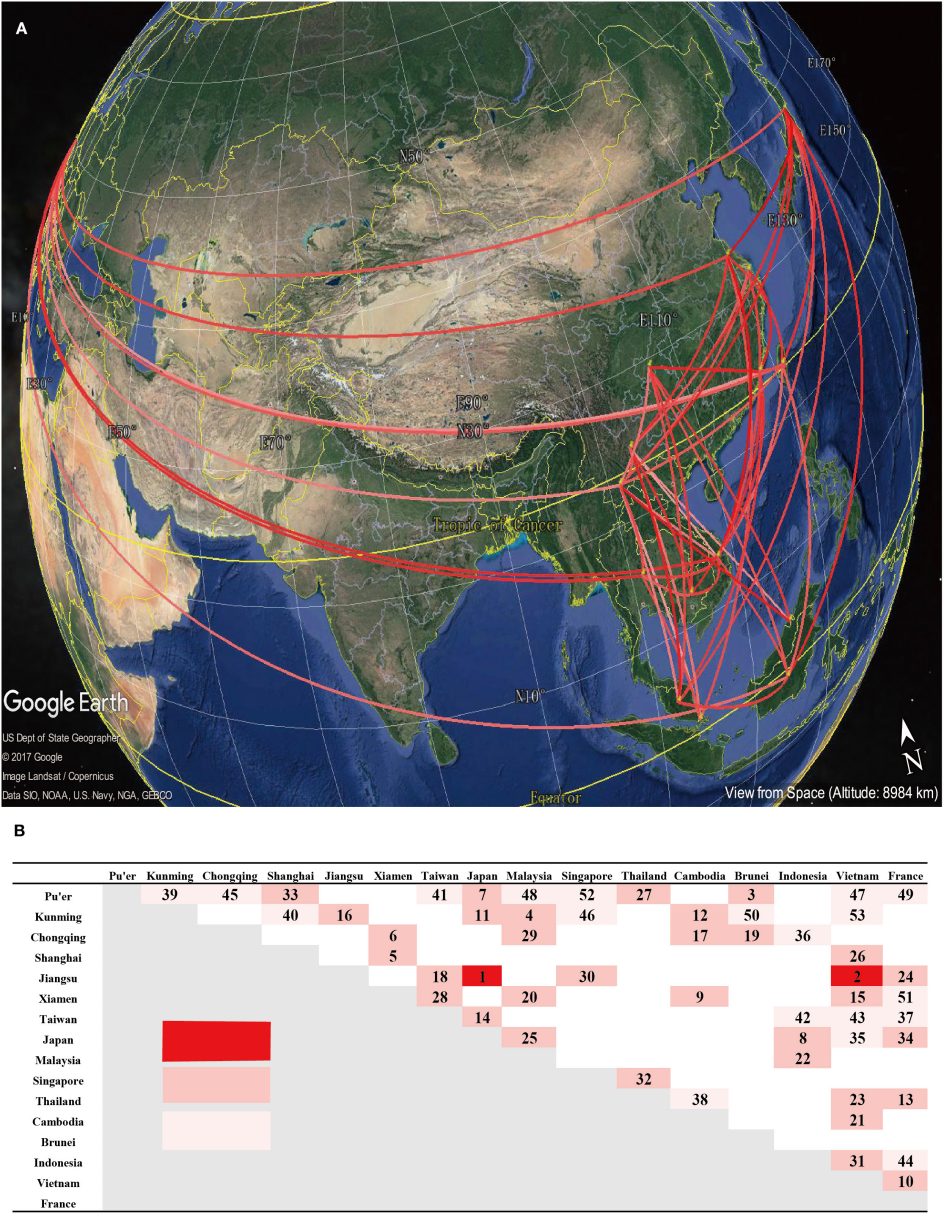
Estimation of the geographical origin and spread of the B5 subgenotype of EV-A71. **(A)** Spatial spread links of B5 sub-genotype of EV-A71 among sampling locations were identified. The lines connecting different locations are colored according to the heat map. **(B)** The heat map shows Bayes factor (BF) values estimated between two geographic locations. The maps are based on satellite pictures made available in Google Earth (http://earth.google.com).

In order to specify significant connections, while comparing prior and posterior probabilities, we summarized the BF related to the migration rates to explain the diffusion patterns (mean Poisson Prior = 0.69, offset = 15). At a BF cutoff of 3.0 and a verage posterior value cutoff of 0.5, we observed significant connections (Figure 5). Pu'er City (in Yunnan, China) exhibited the most number of connections with Vietnam, while the strongest connection observed, with a BF of 2,611, occurred between Japan and Jiangsu, China. The several connections presented here, which were inferred by BF values, relatively revealed the transmission dynamics of B5 sub-genotype in several regions. However, more evidences, including the epidemiological investigations, were needed to accurately judge migration events between different regions.

## Discussion

Commonly, classification of enteroviruses postulates that a series of species, clusters, genotypes, subgenotypes, and clades may be defined via genetic distances or the nucleotide differences in the *VP1* region (Brown et al., 1999; Han et al., 2018). The standard method usually results in the correct judgments for the following reasons: (i) Sites on the *VP1* region represent a majority of the antigenic properties. Thus, it is easy to refer to what is already known in order to make necessary comparisons. (ii) Sequences on the *VP1* region have rarely been reported in relation to recombination (Mcwilliam Leitch et al., 2012). By contrast, recombination frequently occurs in the non-structural protein regions, especially in the *3CD* region, and is considered as an important mechanism underlying the enterovirus evolution (Kyriakopoulou et al., 2015; Muslin et al., 2015; Volle et al., 2019). However, the sequences carrying the recombination events are difficult to analyze using regular phylogenetic methods or genetic distance methods, and thus such results can rarely be accepted. (iii) Compared with the genome sequences, it is easier to obtain entire *VP1* sequences. In addition, a large number of sequences provide more choices, thereby preventing data bias. Therefore, the *VP1* sequences were selected to reconstruct migration characteristics of the B5 subgenotype, especially in China.

Posterior probability, an indicator of phylogeography, shows that continuous processes originated in Malaysia, with particular reference to the connections between Malaysia and other countries, such as Japan, Thailand, and Vietnam. We also found population exchanges to be active in those locations, and these may have potentially influenced the viral genotype exchanges. It is consistent with epidemiological evidence indicating that these locations have witnessed many HFMD outbreaks of the B5 subgenotype of EV-A71 in the past years (Mizuta et al., 2014; Nhan et al., 2018; Noisumdaeng et al., 2018, 2019; Van et al., 2019). Besides, posterior probability suggests linkages from Taiwan to Xiamen to Jiangsu within China and from Vietnam to Chongqing city of China, as well as between Thailand, Vietnam, and Yunnan province of China. However, the posterior probability is not what we can speculate. The posterior probability suggests that Pu'er City (in Yunnan, China), became a new hub of infection and formed the only connection to Kunming City (in Yunnan, China), following migration from Thailand. The possibility of spread within mainland of China is very low, which unlike multiple migration events from active roots, may need further investigations. However, surveillance of mainland of China in 2017 has not resulted in any such observations. Equivalently, the two posterior probability-derived results are also debatable. The first concerns the connection between Malaysia and Shanghai, China. Further information is needed regarding this old migration event, as there is little difference between the *VP1* regions of sequences from Shanghai, China, in 2011, and Malaysia, in 2003. The sequence showed a strong strict clock signal which was not due to recombination. On the other hand, discovery of the earliest geographical ancestor and determination of the communication relationships between Malaysia and Singapore depends only on posterior probability, and is unsupported by the Bayesian significance tests. The following two aspects are worth consideration. Extensive research on the B5 subgenotype of EV-A71 was performed only following the outbreak in Sarawak, Malaysia, in 2003. By that time, this subgenotype had stably circulated in Malaysia and even migrated to other countries. However, the typing algorithms, based on distance and nucleotide divergence, are often affected by the number of statistical data, samples, or sequences. The two samples from Malaysia and Singapore, 0815-MAA-00 and 5511/SIN/00, respectively, which were involved in the EV-A71 outbreaks with the B4 subgenotypes, enhanced this flaw (Mcminn et al., 2001; Herrero et al., 2003). However, we have scarcely found any significant connection between Malaysia and Singapore using the BF. Even though posterior probability reveals a potential connection, we have less evidence that the root B5 subgenotype of EV-A71 occurred before the outbreak in Malaysia, in 2003. The only epidemiological proof for circulation of the B4 subgenotype of EV-A71 in the peninsula suggests that it may be related to Singapore or Sarawak, Malaysia. In fact, we may have lost much effective evidence.

In the current study, compared with posterior probability, the BF revealed wide connections, resulting in much interference. This has not been reported in a previous study of the Coxsackievirus A16 (Hassel et al., 2017). Thus, we suggest that it may be related to the number of sequences entering the analysis, the length of target sequences, the similarities of sequences, and the complexity of the spatial processes. Firstly, the divergence of sequences has been found to be generally <8–10%. In addition, compared with the genome sequence, entire *VP1* sequences have fewer dynamic sites or single nucleotide polymorphism sites. This suggests that using genomic sequences may provide more precise processes for tracking the migration events. However, it also indicates the possibility that high frequency recombination may be misleading and may result in conclusions that lead to incredible processes. Secondly, the BF is an algorithm that reveals the significance of connections via the ratio of prior probability to that of the posterior probability. We suggest that this may be likely affected by the number of relatively conserved sequences. When the number of sequences is decreased, some of the low credibility data may disappear. Certainly, we may sometimes lose some roots at the same time. Therefore, in order to explore the migration process accurately, it may be necessary to repeatedly select sequences, not only considering clock signals and population dynamics, but also considering the impact on the posterior probability.

## Data Availability Statement

The datasets generated for this study can be found in the nucleotide sequences of the entire genome for the seven strains determined in this study have been deposited in GenBank nucleotide sequence database under accession numbers MN966512-MN966518.

## Ethics Statement

The studies involving human participants were reviewed and approved by Second Ethics Review Committee of the National Institute for Viral Disease Control and Prevention, Chinese Center for Disease Control and Prevention. The patients/participants provided their written informed consent to participate in this study.

## Author Contributions

KH and YZ conceived and designed the experiments. KH, ZH, and YS performed the experiments. KH, YZ, DY, DW, SZ, and WenbX analyzed the data. KH and YZ wrote the main manuscript. KH prepared all the Tables and Figures. All authors reviewed the manuscript.

## Conflict of Interest

The authors declare that the research was conducted in the absence of any commercial or financial relationships that could be construed as a potential conflict of interest.
